# Supplementary material for: The adaptive landscapes of three global Escherichia coli transcriptional regulators
Source: eLife. 2026 Jul 21;14:RP103774. doi: 10.7554/eLife.103774 (PMC13387746; doi:10.7554/eLife.103774)
Supplement: Supplementary file 1. [file elife-103774-supp1.docx]

**Supplementary File 1. Plasmids used in this study.**

| **Name** | **Antibiotic to select for plasmid persistence (concentration in µg/ml)** | **Relevant features** | **Source** |
| --- | --- | --- | --- |
| pCAW-Sort-Seq-V2 | Chloramphenicol (50) | pBBR1, TetR, sfgfp, mscarlet-I, no TFBS, no TF | This study, derived from ref.^1^ |
| pCAW-Sort-Seq-V2-CRP-CRPᵂᵀ | Chloramphenicol (50) | pBBR1, TetR, sfgfp, mscarlet-I, CRPᵂᵀ operator, crp | This study |
| pCAW-Sort-Seq-V2-CRP-CRP^lib^ | Chloramphenicol (50) | pBBR1, TetR, sfgfp, mscarlet-I, CRP TFBS library, crp | This study |
| pCAW-Sort-Seq-V2-CRP-Neg | Chloramphenicol (50) | pBBR1, TetR, sfgfp, mscarlet-I, promoterless sfgfp, crp | This study |
| pCAW-Sort-Seq-V2-Fis-Fisᵂᵀ | Chloramphenicol (50) | pBBR1, TetR, sfgfp, mscarlet-I, Fisᵂᵀ operator, fis | This study |
| pCAW-Sort-Seq-V2-Fis-Fis^lib^ | Chloramphenicol (50) | pBBR1, TetR, sfgfp, mscarlet-I, promoterless sfgfp, fis | This study |
| pCAW-Sort-Seq-V2-Fis--Neg | Chloramphenicol (50) | pBBR1, TetR, sfgfp, mscarlet-I, Fis TFBS library, fis | This study |
| pCAW-Sort-Seq-V2-IHF-IHFᵂᵀ | Chloramphenicol (50) | pBBR1, TetR, sfgfp, mscarlet-I, IHFᵂᵀ operator, ihf | This study |
| pCAW-Sort-Seq-V2-IHF-IHF^lib^ | Chloramphenicol (50) | pBBR1, TetR, sfgfp, mscarlet-I, IHF TFBS library, ihf | This study |
| pCAW-Sort-Seq-V2-IHF-Neg | Chloramphenicol (50) | pBBR1, TetR, sfgfp, mscarlet-I, promoterless sfgfp, ihf | This study |

**References:**

1. Westmann, C. A., Goldbach, L. & Wagner, A. The highly rugged yet navigable regulatory landscape of the bacterial transcription factor TetR. *Nature Communications* **15**, (2024).
