# Supplementary material for: The adaptive landscapes of three global Escherichia coli transcriptional regulators
Source: eLife. 2026 Jul 21;14:RP103774. doi: 10.7554/eLife.103774 (PMC13387746; doi:10.7554/eLife.103774)
Supplement: Supplementary file 2. [file elife-103774-supp2.docx]

**Supplementary File 2. Strains used in this study.**

| **Strain** | **Genotype** | **Antibiotic resistance** | **Reference** |
| --- | --- | --- | --- |
| SIG10-MAX from Sigma Aldrich (cloning strain) | F- mcrA Δ(mrr-hsdRMS-mcrBC) endA1 recA1 Φ80dlacZΔM15 ΔlacX74 araD139 Δ(ara,leu)7697 galU galK rpsL nupG λ- tonA (StrR) | Streptomycin | Sigma Aldrich |
| E. coli DH5α (cloning strain) | F‑ endA1 glnV44 thi-1 recA1 relA1 gyrA96 deoR nupG Φ80dlacZΔM15 Δ(lacZYA-argF)U169, hsdR17(rK− mK+), λ− | None | ^1^ |
| E. coli JW5702-4 Δcrp mutant strain | Δ(araD-araB)567, ΔlacZ4787(::rrnB-3), λ−, Δcrp-765::kan, rph-1, Δ(rhaD-rhaB)568, hsdR514 | Kanamycin | ^2^ |
| E. coli JW1702-1 ΔihfA mutant strain | Δ(araD-araB)567, ΔlacZ4787(::rrnB-3), λ−, ΔihfA786::kan, rph-1, Δ(rhaD-rhaB)568, hsdR514 | Kanamycin | ^2^ |
| E. coli JW3229-1 Δfis mutant strain | Δ(araD-araB)567, ΔlacZ4787(::rrnB-3), λ−, Δfis-779::kan, rph-1, Δ(rhaD-rhaB)568, hsdR514 | Kanamycin | ^2^ |

**References:**

1. Grant, S. G., Jessee, J., Bloom, F. R. & Hanahan, D. Differential plasmid rescue from transgenic mouse DNAs into Escherichia coli methylation-restriction mutants. *Proceedings of the National Academy of Sciences* 87, 4645–4649 (1990).

2. Baba, T. *et al.* Construction of Escherichia coli K-12 in-frame, single-gene knockout mutants: The Keio collection. *Mol. Syst. Biol.* 2, 2006.0008 (2006).
