# Supplementary material for: The adaptive landscapes of three global Escherichia coli transcriptional regulators
Source: eLife. 2026 Jul 21;14:RP103774. doi: 10.7554/eLife.103774 (PMC13387746; doi:10.7554/eLife.103774)
Supplement: Supplementary file 3. [file elife-103774-supp3.docx]

**Supplementary File 3. Libraries used in the present study.**

| **Sequence Name** | **Sequence*** | **TF** | **Library size** |
| --- | --- | --- | --- |
| CRP | TGTNNTCTAGANNNNNTNTT | CRP-Fis | 4⁸ = 65,536 |
| Fis | NCTCNTTNNNNAAGNNA | CRP-IHF | 4⁸ = 65,536 |
| IHF | TNNNNNATGAGNNN | Fis-IHF | 4⁸ = 65,536 |

*IUPAC nucleotide nomenclature for degenerate nucleotides: N: A, C, T or G.
