# Supplementary material for: The adaptive landscapes of three global Escherichia coli transcriptional regulators
Source: eLife. 2026 Jul 21;14:RP103774. doi: 10.7554/eLife.103774 (PMC13387746; doi:10.7554/eLife.103774)
Supplement: Supplementary file 4. [file elife-103774-supp4.docx]

**Supplementary File 4. TFBS reference (“wild-type”, WT) sequences used in the present study.**

| **Sequence Name** | **Sequence** | **TF** | **Reference** |
| --- | --- | --- | --- |
| WT_CRP_ | TGTGATCTAGATCACATTTT | CRP | ^1,2^ |
| WT_Fis_ | GCTCATTTTTTAAGCAA | Fis | ^3–5^ |
| WT_IHF_ | TAGTTTATGAGGTA | IHF | ^6,7^ |

1. Ebright, R. H., Ebright, Y. W. & Gunasekera, A. Consensus DNA site for the Escherichia coli catabolite gene activator protein (CAP): CAP exhibits a 450-fold higher affinity for the consensus DNA site than for the E.coli lac DNA site. *Nucleic Acids Res.* **17**, 10295–10305 (1989).

2. Gunasekera, A., Ebright, Y. W. & Ebright, R. H. DNA sequence determinants for binding of the Escherichia coli catabolite gene activator protein. *Journal of Biological Chemistry* **267**, 14713–14720 (1992).

3. Shao, Y., Feldman-Cohen, L. S. & Osuna, R. Functional Characterization of the Escherichia coli Fis-DNA Binding Sequence. *J. Mol. Biol.* **376**, 771–785 (2008).

4. Shao, Y., Feldman-Cohen, L. S. & Osuna, R. Biochemical Identification of Base and Phosphate Contacts between Fis and a High-Affinity DNA Binding Site. *J. Mol. Biol.* **380**, 327–339 (2008).

5. Kahramanoglou, C. *et al.* Direct and indirect effects of H-NS and Fis on global gene expression control in Escherichia coli. *Nucleic Acids Res.* **39**, 2073–2091 (2011).

6. Huo, Y. X. *et al.* IHF-binding sites inhibit DNA loop formation and transcription initiation. *Nucleic Acids Res.* **37**, 3878–3886 (2009).

7. Ho, S. Characteristics of IHF Binding to Holliday Junction DNA. (Wesleyan University, Middletown, CT, 2013). doi:10.14418/wes01.1.932.
