# Supplementary material for: The adaptive landscapes of three global Escherichia coli transcriptional regulators
Source: eLife. 2026 Jul 21;14:RP103774. doi: 10.7554/eLife.103774 (PMC13387746; doi:10.7554/eLife.103774)
Supplement: Supplementary file 5. [file elife-103774-supp5.docx]

**Supplementary File 5. Global landscape properties.**

|  | **Number of genotypes¹** | **Number of peaks²** | **Number of low peaks³** | **Number of high peaks⁴** | **Number of squares⁵** | **Magnitude epistasis or additivity⁶˙⁷** | **Simple sign epistasis⁷** | **Reciprocal sign epistasis⁷** |
| --- | --- | --- | --- | --- | --- | --- | --- | --- |
| CRP | 31,975 (49%)¹ | 2,154 (7%) | 2,093 (97.2%) | 61 (2.8%) | 580,289 | 37% | 34% | 28% |
| Fis | 43,222 (66%)¹ | 2,312 (5%) | 2,140 (92.6%) | 172 (7.4%) | 1,194,144 | 34% | 34% | 32% |
| IHF | 41,325 (63%)¹ | 2,453 (6%) | 2,254 (91.9%) | 199 (8.1%) | 1,026,818 | 34% | 33% | 32% |

¹ Percentages indicate the proportion of the studied library relative to its expected size.

² Percentages indicate the proportion of peaks relative to the total number of genotypes.

³ Low peaks are peaks with regulation strengths below the wild-type sequence. Percentages refer to the proportion of all peak genotypes.

⁴ High peaks are peaks with regulation strengths above the wild-type sequence. Percentages refer to the proportion of all peak genotypes.

⁵ A square represents a quadruplet of sequences containing a focal sequence (ab), one double mutant of this sequence (AB), and the two intermediate single mutants (Ab and aB).

⁶ This category includes both magnitude epistasis and additivity (no epistasis) without distinguishing them, because neither of the two subcategories affects peak accessibility^1^.

⁷ Percentages refer to the proportion of all squares.

**References**

1. Aguilar-Rodríguez, J., Payne, J. L. & Wagner, A. A thousand empirical adaptive landscapes and their navigability. *Nat. Ecol. Evol.* 1, 0045 (2017).
