# Supplementary material for: The adaptive landscapes of three global Escherichia coli transcriptional regulators
Source: eLife. 2026 Jul 21;14:RP103774. doi: 10.7554/eLife.103774 (PMC13387746; doi:10.7554/eLife.103774)
Supplement: Supplementary file 6. [file elife-103774-supp6.docx]

**Supplementary File 6. Primers for engineering the pCAW-Sort-Seq-V2 plasmid and for cloning libraries.**

| **Name** | **Sequence** | **Usage** |
| --- | --- | --- |
| pCAW_frag1_F | 5’-CGTCCGACTTACGGAAGGTAGATtttacggc-3’ | Linearizing the pCAW-Sort-Seq fragment 1 for Gibson Assembly |
| pCAW_frag1_R | 5’-CTCGTGCCTAACGGAAGGTAGATtttacggc-3’ | Linearizing the pCAW-Sort-Seq fragment 1 for Gibson Assembly |
| pCAW_frag2_F | 5’-TAAGATTGCCACGGAAGGTAGATtttacggc-3’ | Linearizing the pCAW-Sort-Seq fragment 2 for Gibson Assembly |
| pCAW_frag2_R | 5’-AGGCCTGACTACGGAAGGTAGATtttacggc-3’ | Linearizing the pCAW-Sort-Seq fragment 2 for Gibson Assembly |
| Ultramer_ds_F | 5’-TTCTCAAAAGCTTCCTGCAGTATTC-3’ | Amplifying Ultramer® libraries |
| Ultramer_ds_R | 5’-cggaaagcacatccggtgac-3’ | Amplifying Ultramer® libraries |
| TFBS_R | 5’-CCGTTTGTAGCATCACCTTC-3’ | Sequencing the TFBS region |
| pCAW_Gibs_Lib_F | 5’-gtctgatgagtccgtgaggacg-3’ | Linearizing the pCAW-Sort-Seq plasmid |
| pCAW_Gibs_Lib_R | 5’-GAGAAAAGAAAACCGCCGATCCTG-3’ | Linearizing the pCAW-Sort-Seq plasmid |
| Ultramer_Gibs_F | 5’-GGTGGACAGGATCGGCGGTTTTCTTTTCTCTTCTCAAAAGCTTCCTGCAGTATTC-3’ | Amplifying Ultramer® libraries for Gibson Assembly |
| Ultramer_Gibs_R | 5’-ggctgtttcgtcctcacggactcatcagaccggaaagcacatccggtg-3’ | Amplifying Ultramer® libraries for Gibson Assembly |
