# Supplementary material for: The adaptive landscapes of three global Escherichia coli transcriptional regulators
Source: eLife. 2026 Jul 21;14:RP103774. doi: 10.7554/eLife.103774 (PMC13387746; doi:10.7554/eLife.103774)
Supplement: MDAR checklist [file elife-103774-mdarchecklist1.docx]

**Materials Design Analysis Reporting (MDAR) Checklist for Authors**

*Manuscript: “The adaptive landscapes of three global Escherichia coli transcriptional regulators” (Westmann, Goldbach & Wagner). Submitted to eLife.*

The MDAR framework (https://osf.io/xfpn4/) establishes a minimum set of requirements for transparent reporting in the life sciences. For all items that apply, the table notes where in the article the information is provided; items marked N/A do not apply to this study.

**Materials**

| **Item** | **Indicate where provided (section / figure legend / submission form)** | **N/A** |
| --- | --- | --- |
| **Newly created materials.** *Materials availability statement disclosing availability of newly created materials and any access restrictions.* | Materials and Methods ('Strains and plasmids'; 'Code Availability and Data Analysis').  All newly created plasmids, strains, primers and TFBS libraries are listed in Supplementary Files 1–4 and 6 and deposited at Zenodo (DOI: 10.5281/zenodo.13838265) and the associated GitHub repository. No access restrictions. |  |
| **Antibodies.** *For commercial reagents, provide supplier name, catalogue number and RRID if available.* | No antibodies were used in this study. | X |
| **DNA and RNA sequences.** *Short novel DNA/RNA (primers, probes) should be included or deposited in a public repository.* | Materials and Methods; Supplementary Files: TFBS libraries (Supplementary File 3), reference TFBS (Supplementary File 4) and all primers (Supplementary File 6).  Raw sequencing data: NCBI BioProject PRJNA1162449. Plasmid and primer sequences: Zenodo DOI 10.5281/zenodo.13838265. |  |
| **Cell materials.** *Cell lines / primary cultures: provide species, strain, accession or supplier/catalogue/clone number or RRID.* | No eukaryotic cell lines or primary cultures were used. Bacterial strains are reported under 'Plants and microbes'. | X |
| **Experimental animals.** *Laboratory/model organisms or field animals: provide species, strain, sex, age, genetic modification status.* | No experimental animals were used. | X |
| **Plants and microbes.** *Microbes: provide species and strain, unique accession number if available, and source.* | Microbes — Materials and Methods ('Strains and plasmids') and Supplementary File 2: Escherichia coli SIG10-MAX (Sigma-Aldrich CMC0004); E. coli BW25113-derived KEIO single-gene deletions Δcrp (JW5702-4), ΔihfA (JW1702-1) and Δfis (JW3229-1), obtained from the KEIO collection.  Plants: not applicable (no plants used). |  |
| **Human research participants.** *If collected, report age, sex, gender and ethnicity for all participants (within privacy constraints).* | No human research participants were involved. | X |

**Design**

| **Item** | **Indicate where provided (section / figure legend / submission form)** | **N/A** |
| --- | --- | --- |
| **Study protocol.** *If pre-registered, provide DOI. For clinical trials, provide trial registration number or DOI.* | Not a pre-registered study or clinical trial. | X |
| **Laboratory protocol.** *Provide DOI or other citation if detailed step-by-step protocols are available.* | Detailed step-by-step procedures are provided in Materials and Methods and in Appendix 1 (General procedures; plasmid design and construction; library design, synthesis and cloning; cell sorting; DNA extraction and sequencing; data analysis).  General procedures build on the previously published system of Westmann et al. (cited reference). |  |
| **Sample-size determination.** *State whether/how performed (for in vivo studies).* | Appendix 1 ('Analysing and sorting cells'; 'DNA extraction and sequencing'): rationale for cells sorted per sequence (≥30 cells/sequence; ~100× library coverage; 6,553,600 cells sorted) and required sequencing reads (~30 reads/genotype). |  |
| **Randomisation.** *State whether/how performed (for in vivo studies).* | Not applicable (no in vivo animal experiments; comprehensive TFBS libraries assayed). | X |
| **Blinding.** *State whether/how performed (for in vivo studies).* | Not applicable (high-throughput automated sort-seq measurements). | X |
| **Inclusion / exclusion criteria.** *State whether/how performed (for in vivo studies).* | Materials and Methods ('Regulation strengths') and Appendix 1 (Data analysis 7.3): pre-established quality filters — variants must appear in all three replicates, have ≥30 total reads, and a coefficient of variation ≤0.5. |  |
| **Sample definition and in-laboratory replication.** *State number of times the experiment was replicated; define technical vs biological replicates.* | Three independent biological replicates per transcription factor (independent library transformations/cultures); plate-reader validation in biological and technical triplicates (three colonies per sample, three wells per colony).  Materials and Methods ('Sort-Seq procedure'; 'Validating regulation strengths with plate reader measurements') and figure legends (Figure 1—figure supplements 4–9 and Figure 2—figure supplement 2). |  |
| **Ethics.** *Human / animal / field studies: state authority granting ethics approval and reference number.* | Microbial laboratory study; no ethics approval required. | X |
| **Dual Use Research of Concern (DURC).** *If subject to DURC regulations, state approving authority and reference number.* | Not applicable. | X |

**Analysis**

| **Item** | **Indicate where provided (section / figure legend / submission form)** | **N/A** |
| --- | --- | --- |
| **Attrition.** *Describe whether exclusion criteria were pre-established; report omitted samples/data points and justification.* | Materials and Methods ('Regulation strengths'), Appendix 1 (Data analysis 7.3) and Results. Exclusion criteria were pre-established. Genotypes were excluded for low read coverage (<30 reads), absence from any of the three replicates, or CV>0.5.  Sequence coverage was reduced from 95/90/93% to 49/66/63% of the library (CRP/Fis/IHF) after quality filtering. |  |
| **Statistics.** *Describe statistical tests used and justify the choice of tests.* | Figure legends and Materials and Methods / Appendix 1. Tests include Welch's two-sample t-tests, Pearson and Spearman correlation tests, and one-sided Monte Carlo permutation tests. Test statistics, degrees of freedom, sample sizes and p-values are reported in the relevant figure legends and in Supplementary File 5. |  |
| **Data availability.** *Manuscript includes a data availability statement; provide accession/DOI and licensing for new datasets and reused data.* | Data availability statement. Sequencing data: NCBI BioProject PRJNA1162449. Processed data, plasmid and primer sequences: Zenodo DOI 10.5281/zenodo.13838265. |  |
| **Code availability.** *Provide details for access to code essential for replicating the main findings (accession/DOI/URL and licensing).* | Code availability statement. All code for data processing, analysis and plotting: Zenodo DOI 10.5281/zenodo.13838265 and the associated GitHub repository. |  |

**Reporting**

| **Item** | **Indicate where provided (section / figure legend / submission form)** | **N/A** |
| --- | --- | --- |
| **Adherence to community standards.** *State if relevant guidelines (ICMJE, MIBBI, ARRIVE, STRANGE) were followed and whether a checklist (CONSORT, PRISMA, ARRIVE) is provided.* | No discipline-specific reporting checklist (e.g., ARRIVE, CONSORT, PRISMA, STRANGE) is applicable to this microbial molecular-biology study. This completed MDAR checklist is provided with the manuscript. |  |
