# Supplementary material for: The adaptive landscapes of three global Escherichia coli transcriptional regulators
Source: eLife. 2026 Jul 21;14:RP103774. doi: 10.7554/eLife.103774 (PMC13387746; doi:10.7554/eLife.103774)
Supplement: Peer review history

# Editor report: eLife Assessment

**DOI:** 10.7554/eLife.103774.3.sa0

**Authors:** Vincent J Lynch

**Affiliations:** University at Buffalo, State University of New York United States

**Keywords:** Convincing, Important

This study maps the genotype-phenotype landscapes of three *E. coli* transcription factors and the topographical features of these landscapes. It shows that ruggedness and epistasis do not hinder the evolution of strong transcription factor binding sites. These **convincing** findings contribute **important** insights into fitness landscape theories and highlight the role of chance, contingency, and evolutionary biases in gene regulation. The authors then study the topographical features of these landscapes, especially the number and distribution of local maxima, as well as the statistical properties of evolutionary paths on these landscapes.

# Referee report: Reviewer #1 (Public review)

**DOI:** 10.7554/eLife.103774.3.sa1

Summary:

For each of three key transcription factor (TF) proteins in *E. coli*, the authors generate a large library of TF binding site (TFBS) sequences on plasmids, such that each TFBS is coupled to the expression of a fluorescence reporter. By sorting the fluorescence of individual cells and sequencing their plasmids to identify each cell's TFBS sequence (sort-seq), they are able to map the landscape of these TFBSs to the gene expression level they regulate. The authors then study the topographical features of these landscapes, especially the number and distribution of local maxima, as well as the statistical properties of evolutionary paths on these landscapes. They find the landscapes to be highly rugged, with about as many local peaks as a random landscape would have, and with those peaks distributed approximately randomly in sequence space. This is quite different from previous work on landscapes for eukaryotic TFBSs, which tend to be rather smooth. The authors find that there are a number of peaks that produce regulation stronger than that of the wild-type sequence for each TF, and that it is not too unlikely to reach one of those "high peaks" from a random starting sequence. Nevertheless, the basins of attraction for different peaks have significant overlap, which means that chance plays a major role in determining which peak a population will evolve to.

Strengths:

(1) The apparent differences in landscape topography between prokaryotic TFBSs and other molecular landscapes is a fascinating discovery to add to the field of genotype-phenotype maps. I am really excited to learn the molecular mechanisms of this in the future.

(2) The experiments and analysis of this paper are very well-executed and, by and large, very thorough. I appreciated the systematic nature of the project, both the large-scale experiments done on three TFs with replicates, and the systematic analysis of the resulting landscapes. This not only makes the paper easy to follow, but also inspires confidence in their results since there is so much data and so many different ways of analyzing it. It's a great recipe for other studies of genotype-phenotype landscapes to follow.

(3) Considering how technical the project was, I am really impressed at how easy to read I found the paper, and the authors deserve a lot of credit for making it so. They do a great job of building up the experiments and analyses step-by-step, and explaining enough of the basics of the experimental design and essence of each analysis in the main text without getting too complicated with details that can be left to the Methods or SI.

Weaknesses:

(1) Regarding the effect of measurement uncertainties, one way in which they attempt to test their effect is to simulate dynamics on noisy and noise-free versions of the landscape and measure visitation frequencies. While they show that visitation frequencies are highly correlated between these cases, I'd prefer a more direct test of epistasis or navigability (e..g, number of local peaks), since that's how they are characterizing the landscapes, and the connection between that and visitation frequency of individual states is unclear.

(2) I am still a little concerned about the fraction of sequences missing from the data due to filtering, although I appreciate the difficulties in testing the importance of this (requiring additional assumptions) and the authors' good-faith efforts to do their best with the data they have.

# Referee report: Reviewer #2 (Public review)

**DOI:** 10.7554/eLife.103774.3.sa2

The authors aim to investigate the ability of evolution to create strong transcription factor binding sites (TFBSs) de novo in *E. coli*. They focus on three global transcriptional regulators: CRP, Fis, and IHF, using a massively parallel reporter assay to evaluate the regulatory effects of over 30,000 TFBS variants. By analyzing the resulting genotype-phenotype landscapes, they explore the ruggedness, accessibility, and evolutionary dynamics of regulatory landscapes, providing insights into the evolutionary feasibility of strong gene regulation. Their experiments show that de novo adaptive evolution of new gene regulation is feasible. It is also subject to a blend of chance, historical contingency, and evolutionary biases that favor some peaks and evolutionary paths.

(1) Strengths of the methods and results:

The authors successfully employed a well-designed sort-seq assay combined with high-throughput sequencing to map regulatory landscapes. The experimental design ensures reliable measurement of regulation strengths. Their system accounts for gene expression noise and normalizes measurements using appropriate controls.

Comprehensive Landscape Mapping:

The study examines ~30,000 TFBS variants per transcription factor, providing statistically robust and thorough maps of the regulatory landscapes for CRP, Fis, and IHF. The landscapes are rigorously analyzed for ruggedness (e.g., number of peaks) and epistasis, revealing parallels with theoretical uncorrelated random landscapes.

Evolutionary Dynamics Simulations:

Through simulations of adaptive walks under varying population dynamics, the authors demonstrate that high peaks in regulatory landscapes are accessible despite ruggedness. They identify key evolutionary phenomena, such as contingency (multiple paths to peaks) and biases toward specific evolutionary outcomes.

Biological Relevance and Novelty:

The author's work is novel in focusing on global regulators, which differ from previously studied local regulators (e.g., *TetR*). They provide compelling evidence that rugged landscapes are navigable, facilitating de novo evolution of regulatory interactions. The comparison of landscapes for CRP, Fis, and IHF underscores shared topographical features, suggesting general principles of global transcriptional regulation in bacteria.

(2) Weaknesses of the methods and results:

Undersampling of Genotype Space:

Approximately 40% of the theoretical TFBS genotype space remains uncharacterized after quality filtering. The authors now discuss this limitation more explicitly and provide analyses suggesting that undersampling does not strongly bias their conclusions at the landscape level. Nevertheless, predictive modeling approaches could further extend these landscapes in future work.

Simplified Regulatory Architecture:

The study considers a minimal system consisting of a single TFBS upstream of a reporter gene. While this simplification allows clean interpretation and high-throughput measurement, natural promoters often involve combinatorial regulation and chromosomal context effects that may alter landscape topography.

Lack of Experimental Evolution Validation:

The evolutionary conclusions are based on simulations rather than direct experimental evolution. The authors provide a reasonable justification for this choice and frame their conclusions at the statistical level rather than for specific trajectories, but experimental validation would be a valuable future extension.

Impact on the Field:

This study advances our understanding of adaptive landscapes in gene regulation and offers a critical step toward deciphering how global regulators evolve de novo binding sites. The findings provide foundational insights for synthetic biology, evolutionary genetics, and systems biology by highlighting the evolutionary accessibility of strong regulation in bacteria.

Utility of Methods and Data:

The sort-seq approach, combined with landscape analysis, provides a robust framework that can be extended to other transcription factors and systems. If made publicly available, the study's data and code would be valuable for researchers modeling transcriptional regulation or studying evolutionary dynamics.

Additional Context:

The study builds on a growing body of work exploring regulatory evolution. For instance, recent studies on local regulators like *TetR* and AraC have revealed high ruggedness and epistasis in TFBS landscapes. This study distinguishes itself by focusing on global regulators, which are more complex biologically and more influential in bacterial gene networks. The observed evolutionary contingency aligns with findings in other biological systems, such as protein evolution and RNA folding landscapes, underscoring the generality of these evolutionary principles.

Conclusion:

The authors successfully mapped the genotype-phenotype landscapes for three global regulators and simulated evolutionary dynamics to assess the feasibility of strong TFBS evolution. They convincingly demonstrate that ruggedness and epistasis, while prominent, do not preclude the evolution of strong regulation. Their results support the notion that gene regulation evolves through a blend of chance, contingency, and evolutionary biases.

This paper makes a significant contribution to the understanding of regulatory evolution in bacteria. While minor limitations exist, the authors' methods are robust, and their findings are well-supported. The work will likely be of broad interest to researchers in molecular evolution, synthetic biology, and gene regulation.

# Author comment: Author response

**DOI:** 10.7554/eLife.103774.3.sa3

**Authors:** Cauã Antunes Westmann, Leander Goldbach, Andreas Wagner

**Affiliations:** University of Zurich Zürich Switzerland; University of Zurich Zürich Switzerland; University of Zurich Zurich Switzerland

The following is the authors’ response to the original reviews.

**Public Reviews:**

**Reviewer #1 (Public review):**

Weaknesses:

(1) The main weakness of this paper, in my view, is that it felt disconnected from the larger body of work on fitness and genotype-phenotype landscapes, including previous data on TFBSs in *E. coli*, genotype-phenotype maps of TFBSs in other systems, protein sequence landscapes (e.g., from mutational scans or combinatorially-complete libraries), and fitness landscapes of genomic mutations (e.g., combinatorially-complete landscapes of antibiotic resistance alleles). I have no doubt the authors are experts in this literature, and they probably cite most of it already given the enormous number of references. But they don't systematically introduce and summarize what was already known from all that work, and how their present study builds on it, in the Abstract and Introduction, which left me wondering for most of the paper why this project was necessary. Eventually, the authors do address most of these points, but not until the end, in the Discussion. Readers who have no familiarity with this literature might read this paper thinking that it's the first paper ever to study topography and evolutionary paths on genotype-phenotype landscapes, which is not true.

There were two points that made this especially confusing for me. First, in order to choose which nucleotides in the binding sites to vary, the authors invoke existing data on the diversity of these sequences (position-weight matrices from RegulonDB). But since those PWMs can imply a genotype-phenotype map themselves, an obvious question I think the authors needed to have answered right away in the Introduction is why it is insufficient for their question. They only make a brief remark much later in the Results that the PWM data is just observed sequence diversity and doesn't directly reflect the regulation strength of every possible TFBS sequence. But that is too subtle in my opinion, and such a critical motivation for their study that it should be a major point in the Introduction.

The second point where the lack of motivation in the Introduction created confusion for me was that they report enormous levels of sign epistasis in their data, to the point where these landscapes look like random uncorrelated landscapes. That was really surprising to me since it contrasts with other empirical landscape data I'm familiar with. It was only in the Discussion that I found some significant explanation of this - namely that this could be a difference between prokaryotic TFBSs, as this paper studies, and the eukaryotic TFBSs that have been the focus of many (almost all?) previous work. If that is in fact the case - that almost all previous studies have focused on eukaryotic TFBSs or other kinds of landscapes, and this is the first to do a systematic test of prokaryotic TFBS, then that should be a clear point made in the Abstract and Introduction. (I find a comparable statement only in the very last paragraph of the Discussion.) If that's the case, then I would also find that point to be a much stronger, more specific conclusion of this paper to emphasize than the more general result of observing epistasis and contingency (as is currently emphasized in the Abstract), which has been discussed in tons of other papers. This raises all sorts of exciting questions for future studies - why do the landscapes of prokaryotic TFBSs differ so dramatically from almost all the other landscapes we've observed in biology? What does that mean for the evolutionary dynamics of these different systems?

We thank the reviewer for this thoughtful and detailed critique. We agree that the original version of the manuscript did not sufficiently motivate the study early on, nor did it clearly position our work within the broader literature on genotype–phenotype (GP) and fitness landscapes. We also agree that two specific issues, the role of PWMs and the unexpectedly high levels of sign epistasis, were insufficiently explained early on, which could lead to confusion for readers not already familiar with this field.

Positioning within the broader landscape literature

In response, we have substantially revised the Abstract and Introduction to explicitly situate our work within existing empirical studies of GP and fitness landscapes, including TFBS landscapes in bacteria, eukaryotic TFBS genotype–phenotype maps, in vitro TF–DNA binding studies, deep mutational scans of proteins, and combinatorially complete fitness landscapes such as antibiotic resistance alleles (Abstract; Introduction, lines 64–85). We now make clear that our study builds directly on this extensive body of work, rather than introducing the landscape framework itself. For example, we write in the introduction:

“Over the last decade, genotype–phenotype (GP) maps and fitness landscapes have become central tools for understanding how molecular systems evolve under mutation and selection[22–25]. Such maps and landscapes have been experimentally studied for DNA[6,8,18,19,26,27], protein[28–32] and RNA[33–35] molecules, revealing key topographical properties that shape evolutionary outcomes, including epistasis[24,36]—the non-additive effects of multiple mutations on phenotype—landscape ruggedness, reflected in the number and distribution of fitness peaks, and constraints on adaptive evolution.”

At the same time, we clarify what remains rare in the literature: large-scale, in vivo genotype–phenotype landscapes for bacterial transcription factor binding sites that are sufficiently dense to support explicit evolutionary analyses. While numerous high-throughput studies have characterized bacterial regulatory elements, these datasets typically do not provide quantitative regulatory phenotypes across large genotype spaces, nor do they analyze evolutionary accessibility. To our knowledge, only one such in vivo TFBS landscape had previously been characterized at comparable resolution for a bacterial local regulator (*TetR*). Our work extends this approach to three global regulators, enabling systematic comparisons across prokaryotic systems (Abstract, Introduction, lines 64–85). For example, we write in the introduction:

“For transcription factor binding sites, most pertinent large-scale studies are based on in vitro binding assays, such as protein-binding microarrays (PBMs), and they focus predominantly on eukaryotic transcription factors[6]. While these studies have been instrumental in characterizing transcription factor binding preferences, they typically do not measure regulatory output in a native cellular context. In contrast, comprehensive in vivo data for bacterial TFBSs remain extremely rare. To our knowledge, only two high-resolution in vivo landscapes have been previously mapped for bacterial regulators, those of the local regulators *TetR*[18] and LacI[27]. As a result, it remains unclear whether principles inferred from protein landscapes, eukaryotic TFBSs, or in vitro binding assays generalize to transcriptional regulation in bacteria, particularly for global regulators[11] that integrate multiple physiological signals.”

Why PWMs are insufficient for our question.

We agree with the reviewer that our original explanation of the role of PWMs was too cursory and should have been addressed explicitly in the Introduction. We have now revised the Introduction to clearly explain why PWMs derived from RegulonDB cannot substitute for empirical GP landscapes in our study (Introduction, lines 102–113).

In this passage we now explain that, first, PWMs are inferred from a limited number of naturally occurring binding sites—typically on the order of hundreds of sequences—whose diversity reflects evolutionary history and genomic context rather than systematic exploration of sequence space. As a result, PWMs sample only a small and biased subset of the possible TFBS variants, whereas our libraries probe tens of thousands of sequences in a controlled manner, providing substantially broader and more uniform coverage of genotype space (Introduction, lines 102–113).

Second, PWM scores are not direct measurements of regulatory strength. Instead, they represent probabilistic or heuristic scores that are primarily used for identifying candidate binding sites in genomes. Numerous studies have shown that PWM scores often correlate weakly with in vivo binding affinity or regulatory output, where DNA shape, cooperative interactions, and chromosomal context play important roles. As such, PWMs do not provide quantitative genotype–phenotype relationships for regulation strength (Introduction, lines 102–113).

Third, PWMs assume independent and additive contributions of individual nucleotide positions. This assumption excludes epistatic interactions by construction. Because epistasis is central to landscape ruggedness, peak structure, and evolutionary accessibility, PWM-based models are fundamentally unsuited to address the evolutionary questions we study here (Introduction, lines 102–113). We now explicitly state this limitation early in the manuscript, rather than only alluding to it later in the Results.

Sign epistasis and contrast with prior TFBS landscapes.

We also agree with the reviewer that the extensive sign epistasis we observe—approaching levels expected for uncorrelated random landscapes—is surprising in light of much of the existing empirical landscape literature. Importantly, as the reviewer notes, most previous TFBS landscape studies have focused on in vitro binding systems or on eukaryotic transcription factors, which tend to exhibit smoother and more additive landscapes.

To address this concern, we have revised the Abstract and Introduction to explicitly frame this contrast as a central result of the study (Abstract; Introduction, lines 151-153, Discussion, lines 652–668). For example, we write in the discussion:

“We showed that the regulatory landscapes of all three TFs are highly rugged and have multiple peaks. The ruggedness of all three landscapes is also supported by the prevalence of epistasis between pairs of TFBS mutations (Supplementary Table S5). A particularly important form of epistasis is sign epistasis[24,93,94], because it can lead to multiple adaptive peaks [24,93,94] (see Supplementary Methods 7.5). Our landscapes contain up to 65% of mutation pairs with sign epistasis, a value that is especially high compared to the almost exclusively additive interactions of mutations in eukaryotic TFs[6,125].”

We now emphasize that prokaryotic TFBS landscapes, particularly for global regulators, appear to be substantially more rugged and epistatic than most previously characterized TFBS landscapes, and that this difference likely reflects fundamental biological distinctions between regulatory systems.

Revised emphasis and conclusions.

Following the reviewer’s suggestion, we have adjusted the emphasis of the manuscript accordingly. Rather than highlighting epistasis and contingency as generic evolutionary phenomena, we now present the extreme ruggedness of prokaryotic TFBS landscapes as a system-specific finding with important implications for the evolution of gene regulation. We explicitly note that this raises new questions for future work—such as why prokaryotic regulatory landscapes differ so markedly from eukaryotic ones, and how these differences shape evolutionary dynamics—which we now highlight in the Introduction and Discussion (Abstract; Introduction, lines 151-153, Discussion, lines 652–668). For example, we write in the discussion:

“… A possible reason for this greater incidence of epistasis lies in the nature of prokaryotic TFBSs. Specifically, prokaryotic TFBSs are at approximately 20bps twice as long as eukaryotic TFBSs[80,128] and exhibit symmetries that reflect the dimeric state of their cognate TFs[129–131]. These factors may increase the likelihood of intramolecular epistasis. Our observations raise important questions for future work, such as why the landscapes of prokaryotic TFBSs differ so dramatically from those of eukaryotic ones. And what do these differences imply for the evolutionary dynamics of gene regulation?”

We believe that these revisions substantially improve the clarity, motivation, and positioning of the manuscript, and directly address the reviewer’s concerns by making both the necessity and the novelty of the study clear from the outset.

(2) I am a bit concerned about the lack of uncertainties incorporated into the results. The authors acknowledge several key limitations of their approach, including the discreteness of the sort-seq bins in determining possible values of regulation strength, the existence of a large number of unsampled sequences in their genotype space, as well as measurement noise in the fluorescence readouts and sequencing. While the authors acknowledge the existence of these factors, I do not see much attempt to actually incorporate the effect of these uncertainties into their conclusions, which I suspect may be important. For example, given the bin size for the fluorescence in sort-seq, how confident are they that every sequence that appears to be a peak is actually a peak? Is it possible that many of the peak sequences have regulation strengths above all their neighbors but within the uncertainty of the fluorescence, making it possible that it's not really a peak? Perhaps such issues would average out and not change the statistical nature of their results, which are not about claiming that specific sequences are peaks, just how many peaks there are. Nevertheless, I think the lack of this robustness analysis makes the results less convincing than they otherwise would be.

We thank the reviewer for raising this important concern. We fully agree that uncertainties arising from experimental resolution, measurement noise in fluorescence and sequencing, and incomplete sampling of genotype space should be incorporated explicitly into the analysis. While these limitations were acknowledged qualitatively in the original manuscript, we recognize that a direct, quantitative assessment of their impact on our conclusions is essential to strengthen the robustness of the study.

We first clarify that regulation strength is not discretized in our analysis. For each TFBS, regulation strength is calculated as a continuous weighted average of fluorescence across all sorting bins, based on the sequencing read-count distribution of each sequence across bins. We clarified this information in the main text (Results, lines 201-203). Nevertheless, finite binning resolution and experimental noise introduce uncertainty in these estimates, which could in principle affect the identification of local peaks.

Importantly, our study does not aim to assert that specific TFBS sequences are definitively peaks. Rather, our focus is on landscape-level statistical and topological properties—such as ruggedness, the abundance and distribution of peaks, and the evolutionary accessibility of strong regulation. We therefore centered our new analyses on testing whether these conclusions are robust to experimentally plausible sources of uncertainty, rather than on the identity of individual peaks.

To address the reviewer’s concern, we performed two complementary analyses. The first evaluates whether the observed ruggedness of the landscapes could arise as an artifact of incomplete sampling. It addressed the effects of missing genotypes and the possibility of spurious peak identification due to unsampled neighbors. Sparse sampling can introduce opposing biases: true peaks may be missed, while other genotypes may be falsely classified as peaks because fitter neighbors are absent. As shown for uncorrelated random (House-of-Cards) landscapes (Kauffman & Levin, 1987), these effects can partially cancel.

In this analysis, we constructed a null model by randomly permuting regulation strengths across the mapped genotype network while preserving its topology. The number of peaks in these randomized landscapes is only modestly higher than in the empirical data, indicating that the measured landscapes are close to the maximal ruggedness compatible with the sampled network (Results, lines 308–320).

In addition, we quantified potential sampling bias by analyzing genotype connectivity. Here we defined the relative connectivity of a genotype as the fraction of possible single-mutant neighbors for which we had measured regulation strength. We observed only a very weak correlation between connectivity and regulation strength (R=-0.1, -0.1, 0.01 for the CRP, Fis, and IHF landscapes, Figures S13-S15). Similarly, the relative connectivity of peak genotypes is only weakly correlated with their regulation strength (R=-0.05, -0.04, 0.06 for the CRP, Fis, and IHF landscapes). (Results, lines 321–330), indicating that strongly regulating genotypes are not preferentially oversampled or undersampled (Results, lines 321–330).

The second, and most important, analysis directly addresses the reviewer’s concern that experimental uncertainty could affect peak classification and, consequently, landscape navigability. We explicitly incorporated experimentally measured, genotype-specific noise estimates from biological replicates when comparing fitness values between neighboring genotypes. Using these uncertainty-aware comparisons, we then recomputed adaptive-walk dynamics and genotype visitation frequencies on the resulting noisy landscapes.

We observe strong correlations between visitation frequencies in the noise-free and noisy landscapes across all three transcription factors (new Supplementary Figure S35), indicating that evolutionary accessibility patterns are robust to realistic levels of experimental uncertainty. These analyses are described in the revised Results (lines 622–636) and in a new Supplementary Methods section (“Incorporation of experimental uncertainty into adaptive walks”).

**Reviewer #2 (Public review):**

The authors aim to investigate the ability of evolution to create strong transcription factor binding sites (TFBSs) de novo in *E. coli*. They focus on three global transcriptional regulators: CRP, Fis, and IHF, using a massively parallel reporter assay to evaluate the regulatory effects of over 30,000 TFBS variants. By analyzing the resulting genotype-phenotype landscapes, they explore the ruggedness, accessibility, and evolutionary dynamics of regulatory landscapes, providing insights into the evolutionary feasibility of strong gene regulation. Their experiments show that de novo adaptive evolution of new gene regulation is feasible. It is also subject to a blend of chance, historical contingency, and evolutionary biases that favor some peaks and evolutionary paths.

(1) Strengths of the methods and results:

The authors successfully employed a well-designed sort-seq assay combined with high-throughput sequencing to map regulatory landscapes. The experimental design ensures reliable measurement of regulation strengths. Their system accounts for gene expression noise and normalizes measurements using appropriate controls.

Comprehensive Landscape Mapping:

The study examines ~30,000 TFBS variants per transcription factor, providing statistically robust and thorough maps of the regulatory landscapes for CRP, Fis, and IHF. The landscapes are rigorously analyzed for ruggedness (e.g., number of peaks) and epistasis, revealing parallels with theoretical uncorrelated random landscapes.

Evolutionary Dynamics Simulations:

Through simulations of adaptive walks under varying population dynamics, the authors demonstrate that high peaks in regulatory landscapes are accessible despite ruggedness. They identify key evolutionary phenomena, such as contingency (multiple paths to peaks) and biases toward specific evolutionary outcomes.

Biological Relevance and Novelty:

The author's work is novel in focusing on global regulators, which differ from previously studied local regulators (e.g., *TetR*). They provide compelling evidence that rugged landscapes are navigable, facilitating de novo evolution of regulatory interactions. The comparison of landscapes for CRP, Fis, and IHF underscores shared topographical features, suggesting general principles of global transcriptional regulation in bacteria.

(2) Weaknesses of the methods and results:

Undersampling of Genotype Space:

While the quality filtering of the data ensures robustness, ~40% of the TFBS space remains uncharacterized. The authors acknowledge this limitation but could improve the analysis by employing subsampling or predictive modeling.

We thank the reviewer for raising this point. We agree that undersampling of genotype space is an important limitation of our dataset and that, in principle, subsampling or predictive modeling approaches could be used to address missing genotypes. We have now clarified in the manuscript why these approaches are not straightforward in the context of our analyses and why we did not pursue them here.

Although approximately 40% of TFBS genotypes were removed during the filtering step due to lack of reliable measurements, this filtering step was necessary to ensure robust estimation of regulation strength from sort-seq data. Importantly, random subsampling of the genotypes in our data set would not alleviate this limitation, because many of our key analyses—such as peak identification, quantification of epistasis, and assessment of evolutionary accessibility—require combinatorially complete local neighborhoods in genotype space. Subsampling would remove mutational neighbors from many neighborhoods, and thus further limit our ability to characterize landscape topology.

Predictive modeling approaches could, in principle, be used to infer missing genotypes and reconstruct more complete landscapes. However, developing, experimentally validating, and benchmarking such models would not only substantially expand the scope of an already long paper, it would also require additional assumptions about genotype–phenotype relationships that entail their own limitations. Our primary goal in this work was to provide the first large-scale empirical in vivo regulatory landscapes for global bacterial transcription factors, comprising tens of thousands of experimentally measured variants. We view these empirical landscapes as a necessary foundation upon which predictive modeling and landscape completion can be built in future, complementary studies.

We have now revised the Discussion (lines 760-770) to explicitly articulate these points and to clarify that, while undersampling remains a limitation, it does not invalidate the landscape-level conclusions we draw from the combinatorially complete neighborhoods present in our data. There we also outline predictive modeling as an important directions for future work.

For a more detailed answer regarding subsampling and peak classification, please also see our response to comment (2) of Reviewer #1.

Simplified Regulatory Architecture:

The study considers a minimal system of a single TFBS upstream of a reporter gene. While this may have been necessary for clarity, this simplification may not reflect the combinatorial complexity of transcriptional regulation in vivo.

Point well taken. We have added paragraph to state explicitly that the system we use to study gene regulation is much simpler than most in vivo regulatory circuits (Discussion, lines 797-802)

Lack of Experimental Validation of Simulations:

The adaptive walks are based on simulated dynamics rather than experimental evolution. Incorporating in vivo experimental evolution studies would strengthen the conclusions. Although this is a large request for the paper, that would not prevent publication.

We thank the reviewer for this important point. We fully agree that in vivo experimental evolution would provide a valuable and complementary way to validate the evolutionary dynamics inferred from our simulations. However, we ask for the reviewer's understanding that adding experimental evolution to an (already long) paper would go far beyond the scope of our study.

Also, the goal of our study was not to reproduce evolutionary trajectories experimentally, but to characterize the structure of large empirical regulatory landscapes, and to use these landscapes as a data-driven basis for exploring evolutionary accessibility under well-defined population-genetic assumptions. The adaptive walks we employ are parameterized directly from experimentally measured genotype–phenotype maps, and incorporate established fixation probabilities. Such walks have been widely used to study evolutionary dynamics on empirical landscapes when experimental evolution is not tractable, because it would involve tens of thousands of genotypes that represent small mutational targets and would thus take a long time to evolve.

An additional issue related to the feasibility of experimental evolution is that performing in vivo experimental evolution for the regulatory landscapes analyzed here would require tracking large populations across a combinatorially vast TFBS space, while simultaneously measuring regulatory phenotypes for thousands of evolving lineages, which is currently not experimentally feasible. This is another reason why simulation-based approaches have been the standard method for linking large-scale empirical landscapes to evolutionary dynamics in both theoretical and experimental studies.

Furthermore, our conclusions are intentionally framed at the level of statistical and landscape-wide properties (e.g., accessibility of high peaks, contingency, and evolutionary bias), rather than at the level of specific mutational trajectories. As such, they do not rely on the precise reproduction of any single evolutionary path, but on aggregate patterns that are robust to reasonable variation in population-genetic parameters.

In sum, we do not view experimental evolution as essential for the conclusions we draw, but as an important and exciting direction for future work that may be enabled by the landscapes we have experimentally mapped.

Impact on the Field:

This study advances our understanding of adaptive landscapes in gene regulation and offers a critical step toward deciphering how global regulators evolve de novo binding sites. The findings provide foundational insights for synthetic biology, evolutionary genetics, and systems biology by highlighting the evolutionary accessibility of strong regulation in bacteria.

Utility of Methods and Dat

The sort-seq approach, combined with landscape analysis, provides a robust framework that can be extended to other transcription factors and systems. If made publicly available, the study's data and code would be valuable for researchers modeling transcriptional regulation or studying evolutionary dynamics.

Additional Context:

The study builds on a growing body of work exploring regulatory evolution. For instance, recent studies on local regulators like *TetR* and *AraC* have revealed high ruggedness and epistasis in TFBS landscapes. This study distinguishes itself by focusing on global regulators, which are more biologically complex and influential in bacterial gene networks. The observed evolutionary contingency aligns with findings in other biological systems, such as protein evolution and RNA folding landscapes, underscoring the generality of these evolutionary principles.

Conclusion:

The authors successfully mapped the genotype-phenotype landscapes for three global regulators and simulated evolutionary dynamics to assess the feasibility of strong TFBS evolution. They convincingly demonstrate that ruggedness and epistasis, while prominent, do not preclude the evolution of strong regulation. Their results support the notion that gene regulation evolves through a blend of chance, contingency, and evolutionary biases.

This paper makes a significant contribution to the understanding of regulatory evolution in bacteria. While minor limitations exist, the authors' methods are robust, and their findings are well-supported. The work will likely be of broad interest to researchers in molecular evolution, synthetic biology, and gene regulation.

We thank the reviewer for their thorough evaluation and for their supportive opinion of this paper.

**Recommendations for the authors:**

**Reviewer #1 (Recommendations for the authors):**

(1) Line 28 (Abstract): "Landscape ruggedness does not prevent the evolution of strong regulation, because more than 10% of evolving populations can attain one of the highest peaks." I did not find this interpretation very convincing; only 10% of populations being able to achieve strong regulation sounds to me like ruggedness DOES impede adaptation in the vast majority of cases.

We thank the reviewer for this thoughtful comment and agree that our original phrasing in the Abstract overstated this conclusion. We did not intend to imply that landscape ruggedness has only a minor effect on adaptation. On the contrary, our results clearly show that ruggedness strongly constrains evolutionary outcomes and prevents the majority of evolving populations from reaching the globally highest regulatory peaks. We have therefore toned down the wording in both the Abstract and the Discussion (lines 670-679) to reflect this more accurately. For example, in the abstract we now state

“Nonetheless, evolutionary simulations show that ~10% of evolving populations can reach a peak of strong regulation, a proportion that is significantly greater than in comparable random landscapes.”

In the discussion we state:

“… Specifically, our evolutionary simulations show that 10% of populations with a size typical of *E. coli* reach one of the highest peaks. This percentage is significantly higher than in randomized landscapes (Supplementary Methods 9; Supplementary Figure S30)"

Our intended interpretation was more limited: namely, that ruggedness does not fully preclude the evolution of strong regulation. In highly rugged landscapes with extensive sign epistasis—whose topological properties approach those of uncorrelated random landscapes—the a priori expectation is that access to the strongest peaks could be vanishingly rare or effectively impossible under Darwinian evolution. In this context, observing that a non-negligible fraction of populations (on the order of 10%) can reach one of the highest peaks suggests that strong regulation remains evolutionarily attainable, even though it is far from guaranteed.

Motivated by the reviewer’s suggestion, we also added a null-model analysis that makes this point more explicitly and quantitatively. Specifically, we constructed randomized landscapes by permuting regulation-strength values across genotypes while preserving the experimentally sampled genotype network topology and all parameters of the evolutionary simulations (Supplementary Methods 9, “Randomized landscape null model for peak accessibility”). We then repeated the adaptive-walk simulations on these shuffled landscapes. This null model provides an expectation for peak accessibility in landscapes with identical sampling, neighborhood structure, and evolutionary dynamics, but without genotype–phenotype correlations.

Using this null model, we find that the fraction of populations that reach high peaks in the empirical landscapes is substantially higher than expected by chance alone (new Supplementary Figure S30; Results, lines 504–516). Specifically, across the three transcription factors, empirical landscapes exhibit on average a ~3-fold higher accessibility of high regulatory peaks than shuffled landscapes. This comparison does not weaken the conclusion that ruggedness strongly impedes adaptation; rather, it shows that the structure of the measured genotype–phenotype landscapes enables greater accessibility of strong regulation than would be expected in equally rugged but unstructured landscapes.

In response to the reviewer’s concern, we have revised the abstract and main text to avoid the phrase “does not prevent” and to more accurately convey this balance between constraint and accessibility. We now emphasize that ruggedness strongly constrains adaptation, while still allowing access to strong regulatory peaks at rates that exceed null expectations. (Discussion, lines 512-516). For example, in the discussion we state:

“… In sum, rugged regulatory landscapes strongly constrain evolutionary trajectories, yet do not render the evolution of strong regulation vanishingly rare. Instead, strong regulatory phenotypes remain evolutionarily attainable at levels that exceed null expectations, even though they are reached by only a minority of evolving populations.”

We believe that the revised wording, together with the added null-model analysis more faithfully represents our results and strengthens the quantitative interpretation of accessibility in these landscapes.

(2) Line 123: I found the explanation of the plasmid system and the accompanying SI figures (Figures S1 and S2) confusing in terms of how many plasmids there were. In particular, the Figure S1 graphics show the plasmid specifically with CRP but the text in the graphic and in the caption refers to the plasmid pCAW-Sort-Seq-V2 (which, according to Table S1, isn't that just the base plasmid without any TF?). Figure S2 also shows the plasmid with CRP and does specify pCAW-Sort-Seq-V2-CRP-CRP0 in the graphic, but then the caption refers again only to the base plasmid pCAW-Sort-Seq-V2. I recommend the authors clarify these items for readers who might want to reproduce or build upon their system. In particular, I recommend the main text explain more explicitly that they generate three versions of this plasmid (one for each TF), and then on the backgrounds of each of those three plasmids, a whole library with all the binding site variants.

We thank the reviewer for pointing out this lack of clarity. We agree that the original description of the plasmid system and the accompanying Supplementary Figures S1 and S2 could be confusing with respect to how many plasmids were used and how they differ.

To clarify the experimental design, we start from a common backbone plasmid, pCAW-Sort-Seq-V2, which contains all shared regulatory and reporter elements but does not encode any transcription factor. From this backbone, we generated three distinct TF-specific plasmids, each carrying one of the transcription factors studied here—CRP, Fis, or IHF—resulting in pCAW-Sort-Seq-V2-CRP, pCAW-Sort-Seq-V2-Fis, and pCAW-Sort-Seq-V2-IHF. On the background of each TF-specific plasmid, we then constructed a complete library of plasmids containing all variants of the corresponding TF binding site cloned upstream of the reporter gene.

We have revised the main text to explicitly describe this plasmid hierarchy and library construction strategy and to clarify that three TF-specific plasmids were generated prior to TFBS library construction (Results, Landscape mapping section; lines 159–193). In addition, we have redesigned Supplementary Figures S1 and S2 to facilitate understanding of the plasmid system. Specifically, these figures now clearly distinguish between the base plasmid backbone and the TF-specific plasmid derivatives. Also, the plasmid names shown in the graphics and captions are now consistent with those listed in Supplementary Table S1. Upon final publication, we will also deposit the sequences of all plasmids in Addgene to further facilitate reproducibility.

(3) Line 135: Can the authors clarify whether these TFs are essential in these media conditions and, if not, why? I was expecting them to be so given the core functions of these TFs as described in the Introduction, but then Figure S3 appears to show that all knockouts are viable.

We thank the reviewer for raising this important point and apologize for the lack of clarity in the original version of the manuscript. The transcription factors CRP, Fis, and IHF are not essential for viability under the growth conditions used in this study, but they are important for optimal growth and cellular fitness, consistent with their roles as global regulators.

Under our experimental conditions, single-gene knockout strains (Δcrp, Δfis, and Δihf) are viable but exhibit slower growth dynamics compared to the wild-type strain, reflecting impaired regulation of core cellular processes (Supplementary Figure S3). This behavior is consistent with previous work showing that many global transcriptional regulators in *E. coli* are conditionally essential or strongly fitness-affecting, rather than absolutely essential under standard laboratory conditions.

Importantly, while single knockouts remain viable, double mutants involving these global regulators are not viable, indicating substantial functional redundancy and network-level essentiality among global transcription factors. This explains why each TF can be studied individually in isolation, while combinations of deletions cannot be maintained.

We have now clarified this point in the Results section by explicitly stating that the knockout strains show reduced growth rates but reach comparable cell densities during late exponential or early stationary phase, the growth phase at which all measurements were performed (Results, Landscape mapping section; lines 185–193). This clarification reconciles the apparent discrepancy between the biological importance of these transcription factors discussed in the Introduction and the viability of the single-knockout strains shown in Supplementary Figure S3.

(4) Lines 141 and 227: The authors appear to refer to two different citations for different versions of RegulonDB (refs. 47 and 66). Did they actually use both versions for different purposes (if so, why?), or is this a typo?

We thank the reviewer for noticing this inconsistency. We did not use two different versions of RegulonDB. The two separate references were an error. We have now corrected this by using a single, consistent RegulonDB citation in both locations.

(5) Line 166 (Figure 1 caption): I think 2^8 here should be 4^8.

Thank you. We have corrected “2^8^” to “4^8^” in the Figure 1 caption.

(6) Figure 2Are the distributions in Figure 2a (regulation strengths across all TFBSs in the libraries) equivalent to the distributions in Figures S4-S6 (direct fluorescence readout from cell sorting), just transformed from fluorescence to regulation strength? If so I think that would be helpful to clarify, perhaps in the captions to Figures S4-S6 so that it's clear these contain the same information.

No. Figures S4–S6 and Figure 2a do not show the same distributions. Figures S4–S6 display the raw fluorescence distributions obtained from cell sorting, whereas Figure 2a shows regulation strengths (S), which are derived quantities computed from these fluorescence data. Specifically, regulation strength is calculated as a weighted average over fluorescence bins using the sequencing read distribution for each TFBS (see Methods, “Regulation strengths”).

To clarify this relationship, we have revised the main text (lines 201-203 and Figure 1b-c), to explicitly state how regulation strengths (S) were calculated.

(7) Figure 2b: Can the authors label each logo/frequency matrix with its corresponding TF name in the graphic itself? I think this is only implied in the caption.

We have updated Figure 2b to label each sequence logo / frequency matrix directly in the graphic with its corresponding transcription factor name (CRP, Fis, or IHF), in addition to mentioning these names in the caption. This change clarifies the figure and makes the TF identity immediately apparent to the reader.

(8) Lines 290 and 298 (Figure 2 caption): The labels for panels b and c appear to be swapped in the caption.

We thank the reviewer for pointing this out. The labels for panels b and c in the Figure 2 caption were indeed swapped. This has now been corrected.

(9) Line 379: There is a missing period at the end of this line.

We have added the missing period at the end of this line.

(10) Line 400 (Figure 3 caption): There is a missing subtitle for panel c in the caption for this figure (all other panels seem to have bolded subtitles in their captions).

We have added the missing subtitle for panel c in the Figure 3 caption to match the formatting of the other panels.

(11) Line 583: There is a missing period after "Methods 7.5".

We have added the missing period after “Methods 7.5”.

(12) Line 641: "All three landscapes highly rugged" should probably be "All three landscapes are highly rugged".

We have corrected the sentence to read “All three landscapes are highly rugged.”
